# Tanshinone IIA attenuates fluoride-induced spinal cord injury by inhibiting ferroptosis and inflammation

**DOI:** 10.1016/j.heliyon.2024.e40549

**Published:** 2024-11-28

**Authors:** Qingfeng Shen, Shibo Ma, Lingbo Li, Yingpeng Xia

**Affiliations:** a Department of Spine Surgery, Tianjin Union Medical Center, Tianjin, 300121, China; b Department of Orthopedics, Jingzhou Central Hospital, Jingzhou, 434020, Hubei Province, China

**Keywords:** Tanshinone IIA, Spinal cord injury, Fluoride, Ferroptosis

## Abstract

Excessive fluoride exposure can lead to health problems, such as fluorosis and neurotoxicity. However, effective therapeutic strategies for neurofluorosis remain elusive due to a limited understanding of the underlying molecular mechanisms. This study aimed to investigate the effects of Tanshinone IIA on spinal cord injury induced by high-fluoride exposure. To identify dysregulated genes associated with ferroptosis, we conducted an intersection analysis between differentially expressed genes in fluoride-treated HOS cells (GSE70719) and ferroptosis-related genes from the FerrDb database. A rat model of fluoride-induced spinal cord injury was established, revealing evidence of aberrant molecular and structural changes. Furthermore, the study demonstrated that Tanshinone IIA restored the altered expression of nine ferroptosis-related genes, eight fluorosis-related inflammatory indicators, and the observed structural changes. Overall, these findings suggest that Tanshinone IIA therapeutic potential in the treatment of fluoride-induced spinal cord injury by inhibiting ferroptosis and inflammation.

## 1 Introduction

Fluoride levels exceeding safety standards have been detected in groundwater, soil, and agricultural products worldwide. The World Health Organization (WHO), reports that approximately 137 million people globally consume water from fluoride-containing sources, presenting a potential threat to human health and the environment [1,2]. According to available literature, prolonged exposure to high fluoride concentrations can induce cytotoxicity, immune toxicity, and oxidative damage, resulting in fluorosis, including dental and skeletal fluorosis. Additionally, it can also contribute to dysfunction of the endocrine, digestive, and nervous system [[3], [4], [5], [6]]. Fluoride-induced neurological symptoms encompass cognitive impairment, learning and memory deficits, attention deficits, abnormal behavior, psychiatric disorders, and developmental delays in intelligence. Recent studies have suggested an association between excessive fluoride exposure and a lower average IQ in children [7,8]. Various studies have demonstrated that chronic fluorosis-related neurological symptoms are closely related to fluorine-induced oxidative stress, alterations in signal transduction, and apoptosis. These mechanisms warrant further investigation. Furthermore, molecular biomarkers associated with fluorosis including, Mag, GSK3β, P53, MMP9, and caspase-3 ^9,^ as well as inflammatory indicators such as TNF-α, IL-1β, NF-κB [10,11] have been utilized to assess the extent of spinal cord injury in fluorosis-afflicted rats. Despite these findings, our current understanding of the potential pathogenesis of neurofluorosis remains limited, and specific, effective therapeutic interventions remain elusive.

Recently, ferroptosis has garnered significant attention due to its pivotal role in a wide array of biological processes. Ferroptosis is characterized as an iron-dependent programmed, non-apoptotic cell death process induced by oxidative stress [12]. Numerous studies have elucidated the involvement of ferroptosis in neurotoxicity and brain injury, underscoring the potential therapeutic benefits of inhibiting this process [13]. Elevated extracellular glutamate levels have been implicated in the pathogenesis of brain diseases, triggering ferroptosis by inhibiting the function of systemic XC, a central regulator of ferroptosis. Notably, rat hippocampal sections have revealed that excitatory neuronal death induced by glutamate can be blocked by the ferroptosis inhibitor ferrostatin [14]. Ferroptosis has also been found to contribute to injury in animal models, and the inhibition of ferroptosis has been demonstrated to promote neurological recovery. For instance, rats with spinal cord injury exhibited significant changes in ferroptosis markers, coupled with alterations in the mitochondrial characteristics of ferroptosis, as evidenced by transmission electron microscopy [15].

Tanshinone IIA (Tan IIA), a phenanthone derivative, is the primary bioactive compound in salvia miltiorrhiza [16]. Previous studies have demonstrated that Tan IIA possesses anti-inflammatory properties by inhibiting the activation of the NIK-IKK and MAPK (P38, ERK1/2, JNK) signaling pathways [17,18]. Tan IIA has been used for a variety of conditions, including inflammation, platelet aggregation, and cancer [19].In cases of neurological system injury, researchers suggest that Tan IIA shows promise as a therapeutic agent for accelerating peripheral nerve regeneration and regulating apoptosis [[20], [21], [22]]. Additionally, Tan IIA can confer protection against motor dysfunction in zebrafish models of cerebral hemorrhage, the therapeutic mechanism of which is believed to be related to the preservation of vascular integrity and cytoskeletal remodeling [23]. Considering its potential anti-inflammatory and neuroprotective benefits, we intended to explore whether it exerts any effects on fluoride-induced spinal cord injury and investigate its molecular mechanisms, although limited research is available in this field.

In this study, spinal cord injury can be induced by high-fluoride exposure with aberrant molecular and structural indicators. Furthermore, our findings suggested that Tan IIA treatment significantly alleviated spinal cord injury by inhibiting inflammation and ferroptosis. These findings provide valuable insights into the underlying molecular mechanisms of Tan IIA and suggest that it may represent a promising novel therapeutic approach for spinal cord injury induced by fluoride exposure.

## 2 Materials and methods

### 2.1 Microarray data source

To understand the molecular mechanisms of fluoride-induced toxicity, we retrived gene expression profiles (GSE70719) of HOS cells (HOS) exposed to a sublethal concentration of fluoride (8 ppm) for 30 days were retrieved from the GEO database [24]. In order to understand the detrimental effects of fluoride exposure, we analyzed changes in gene expression in HOS cells exposed to fluoride. We aimed to shed light on the potential molecular mechanisms underlying this change. The limma R package was employed to examine the differentially expressed genes (DEGs) in the dataset GSE70719 between the HOS control and fluoride groups. The fold change (FC) of individual genes was calculated, and DEGs of upregulated and downregulated genes were determined based on the criteria of P < 0.05 and |FC| > 1.3, respectively. The Heatmap R package was used to analyze and visualize the hierarchical clustering of DEGs.

### 2.2 Identification of hub genes

Datasets of ferroptosis regulators and markers were retrieved from the FerrDb database(http://www.zhounan.org/ferrdb/legacy/index.html) to intersect with the clusters of DEGs. To annotate the function of these dysregulated genes associated with ferroptosis in fluoride-treated HOS cells, we conducted Kyoto Encyclopedia of Genes and Genomes (KEGG) and Gene Ontology (GO) enrichment analyses using the DAVID database (https://david.ncifcrf.gov/). A *P* value of less than 0.05 was considered statistically significant. Next, we conducted the protein-protein interaction (PPI) network analysis to identify hub genes using REACTOME (https://reactome.org/) and focused on nodes with a higher node degree, closeness centrality, and stress centrality.

### 2.3 Experimental animals and groups

Adult male Wistar albino rats weighing approximately 100–120 g were obtained from Beijing Vital River Laboratory Animal Technology Co. The study was conducted in accordance with the guidelines of the Institutional Animal Care and Use Committee. Experimental animals were acclimated to our laboratory environment for one week, during which time they were housed under a 12/12 h light/dark cycle and maintained at an ambient environment of 22–25 °C. The animals were fed a standard laboratory diet to ensure a consistent nutritional intake throughout the study period.

A total of 24 healthy adult male rats were used in this study, and they were divided into four groups of six rats each. The initial weights of the rats were calculated before the commencement of the treatment period, which was 180 days for all groups. The first group, the control group, received distilled water for 180 days. The second group, the fluoride group, received ddH_2_O with 100 mg/L of sodium fluoride (NaF) for the same period. The third group, referred to as the Tanshinone IIA control group, received ddH_2_O and Tanshinone IIA at a dose of 20 mg/(kg•d) for 180 days. The fourth group, known as the Tanshinone IIA + fluoride group, received NaF ([100 mg/L]) in ddH_2_O for the first 90 days, followed by distilled water with Tanshinone IIA at the same dose for the remaining 90 days. Sodium fluoride (NaF) was obtained from Sigma-Aldrich (St. Louis, Missouri, United States) and Tanshinone IIA was purchased from SolarBio (Beijing, China). These sample sizes were consistent across all experiments conducted in our study to ensure statistical robustness and meet the ethical standards for animal research.

### 2.4 Immunohistochemical staining

To ensure a humane euthanasia procedure, the rats were anesthetized with pentobarbital sodium (60 μg/g body weight) via intraperitoneal injection. The spinal cords were harvested and post-fixed in the same fixative solution for 24 h at 4 °C. The spinal cords were then transferred to 30 % sucrose in 0.1 M PB at 4 °C until the tissues sank. After serial coronal sectioning of 30 μm sections, the sections were dehydrated, rehydrated, and rinsed. Rat spinal cords were permeabilized with 0.2 % Triton X-100 and blocked (RT) with 5 % goat serum at room temperature for 30 min. Subsequently, the sections were incubated overnight at 4 °C with anti-TNF-α antibodies (1:100, ABclonal, Wuhan, China) and anti-MBP antibodies (1:100, ABclonal, Wuhan, China), respectively. The cells were then incubated with goat anti-rabbit IgG-HRP Secondary Antibody (ab6721; Abcam) (diluted 1:200 in PBST) at room temperature for 2 h. The sections were counted using Image-Pro Plus software (USA).

### 2.5 Transmission electron microscopy

The rats were anesthetized, and fibrous myelin from the spinal cord injuries was perfused with 200 ml of PBS (0.0. M, pH 7.4). To prevent mechanical damage to the tissues, such as pulling, contusion, or extrusion, we limited the tissue volume to no more than 1 mm-cube. The fixative solution used for electron microscopy was added to tissues immediately after collection to initiate fixation, and the samples were kept at 4 °C for a period of 2–4 h. Afterwards, the fibrous myelin of the post-fixed spinal cord injury was osmicated at room temperature for 2 h, rinsed with PBS, and then dehydrated with different concentrations of alcohol (50 %, 70 %, 80 %,90 %,95 %, 100 % and 100 %) for 15min each. The tissues were then embedded at 60 °C for 48 h and subsequently sectioned into ultrathin slices (60–80 nm) using an ultramicrotome from Leica, Germany. Thin sections were collected on grids and stained with uranyl acetate (2 %) and lead citrate for 15 min. Finally, an HT7700 transmission electron microscope (Hitachi, Tokyo, Japan) was used to examine the grids at 80 kV.

### 2.6 Quantitative reverse transcription polymerase chain reaction (RT-qPCR)

Total RNA was extracted from the spinal cord tissues of rats in the four groups using TRIzol reagent (Invitrogen) according to the manufacturer's instructions. RNA concentration was determined using a Nanodrop 2000 spectrophotometer (Mettler Toledo International Trade Co. Ltd., Shanghai, China). Subsequently, 1.0 μg of RNA was reverse transcribed to cDNA using M-ML reverse transcriptase (Takara, Beijing, China). For qPCR, SYBR® Premix Ex™ Taq (Takara) was employed, with GAPDH serving as the reference gene. The primers used for qPCR analysis are listed in Table 1.

**Table 1:** Primers used in qRT-PCR study.

Genes	Primer
36B4 forward	GAGGAATCAGATGAGGATATGGGA
36B4 reverse	AAGCAGGCTGACTTGGTTGC
MMP9 forward	CAAGGATGGTCTACTGGCACACG
MMP9 reverse	AGGTGAAGGGAAAGTGACATGGG
TNF-α forward	GCGTGTTCATCCGTTCTCTACC
TNF-α reverse	TACTTCAGCGTCTCGTGTGTTTCT
IL-1β forward	AGGAGAGACAAGCAACGACA
IL-1β reverse	CTTTTCCATCTTCTTCTTTGGGTAT
MBP forward	TGATGTGTTTGGGGAGGCAGA
MBP reverse	AACCCATAGTTCCTCTACGCC
MAG forward	CCAAGGACGCAGCTTTCTATC
MAG reverse	CTGTGTCAAGGGAATGCTGAAG
P53 forward	TCCTCCCCAA CATCTTATCC
P53 reverse	GCACAAACACGAACCTCAAA
NF-Κb forward	AGGCTCGGAGAGCCCA
NF-Κb reverse	CTGGGGCGGCTGACCGAATG
GSK3β forward	ACCATCCTTATCCCTCCA
GSK3β reverse	CAGAAGCGGCGTTATTG
TFRC forward	GTTGTTGAGGCAGACCTTCA
TFRC reverse	ATGACTGAGATGGCGGAAAC
GDF15 forward	CGGTGAATGGCTCTCAGATG
GDF15 reverse	CAGGTCCTCGTAGCGTTTCC
HMGB1 forward	CAGCAGCAGCACCCGGATGCTTCTGTCAAC
HMGB1 reverse	TGCTGCTGCTGGTGCTCCTCCCGGCAAG
CXCL2 forward	GAAGTCATAGCCACTCTCAAGG
CXCL2 reverse	TTCCGTTGAGGGACAGCA
ACSF2 forward	CCAGTTACGATTTCACGACCA
ACSF2 reverse	TGCCTACACTTCCAGCCTTCT
PCK2 forward	GGAAGCTCCCCAAGTATGAGAACTG
PCK2 reverse	GCTTTCTACCCGTGCCACAT
DDIT4 forward	TAACACCAGGGAGCTGC
DDIT4 reverse	ACAGTTCACTCCTCCAGTACA
FTH1 forward	GCACTGCACTTGGAAAAGAGTGTGAA
FTH1 reverse	CCTGCTCATTCAGGTAATGCGTCT
GPX4 forward	AATCCTGGCCTTCCCTTGCA
GPX4 reverse	GCCCTTGGGCTGGACTTTCA
SLC7A11 forward	AGGGCATACTCCAGAACACG
SLC7A11 reverse	GGGACCAAAGACCTCCAGAA
RRM2 forward	AAGAAGAAGGCTGACTGGGC
RRM2 reverse	ATCAGCCCCCGTTTCTTGAG
NFE2L2 forward	CACTCTGTGGAGTCTTCCATTT
NFE2L2 reverse	GAATGTGTTGGCTGTGCTTTAG
HSPB1 forward	GCACGGCTACATTTCCCGTTGCTTCAC
HSPB1 reverse	TTACTTGTTTTCCGGCTGTTCGGACTTCCC
IREB2 forward	CCTGCTCTTCCCAGACAGTG
IREB2 reverse	ACTGCCTCTGTCTCAATGCC
TXNIP forward	GGACGAGCTCTGAGATGAGC
TXNIP reverse	GAGAGTCGTCCACATCGTCC

### 2.7 Reactive oxygen species (ROS) detection

ROS levels were measured using the CellROX® Deep Red Reagent from Thermo Fisher Scientific, following the manufacturer's protocol. The CellROX® Deep Red reagent was added to each sample to a final concentration of 1000 nM. Samples were then incubated in the dark at 37 °C for 1 h. Following incubation, ROS fluorescence was assessed using the APC channel on a Beckman CytoFLEX LX flow cytometer, with data analysis performed using FlowJo software, version 10.8.

### 2.8 Western blot

Upper thoracic spinal cord tissues from rats were collected and homogenized in RIPA buffer (R002, Solarbio). Protein quantification was performed using the BSA Bradford protein concentration assay kit (AR0145, BOSTER). The proteins were separated by SDS-PAGE (5 % stacking gel and 10 % separating gel) (AR0138, BOSTER) and transferred onto a PVDF membrane (IPVH0010, Millipore). The membranes were then incubated overnight at 4 °C with primary antibodies against CXCL2 (26791-1-AP, Proteintech), PCK2 (67676-1-lg, abcam), RRM2 (DF7248, Affinity), SLC7A11 (BM5318, BOSTER), or β-actin (BA2305, BOSTER). Following washing with TBST buffer, the membranes were incubated with the secondary antibody (ab7090, abcam). Protein bands were visualized using Western LightningTM Chemiluminescence Reagent (NEL10300EA, PerkinElmer) and quantified using the Epson Perfection V39 system (EPSON). Relative protein expression levels were normalized to β-actin using ImageJ software (NIH, Bethesda, MD, USA).

### 2.9 Statistical analysis

The experimental data were repeated three times and presented as the mean ± standard error of the mean (SEM). Unpaired two-tailed Student's t-tests were used to perform statistical analyses between two groups of samples. An analysis of variance (ANOVA) was used to compare the differences among the three groups. If ANOVA showed significant differences among the three groups, the Bonferroni correction was used to determine which groups had significant differences. Statistical significance is indicated by ∗*P* < 0.05, ∗∗*P* < 0.01, and ∗∗∗*P* < 0.001. GraphPad Prism 5 software was used for all the analyses.

## 3 Results

### 3.1 Identifying dysregulated genes related to ferroptosis in fluoride-treated HOS cells

**Fig. 1:**
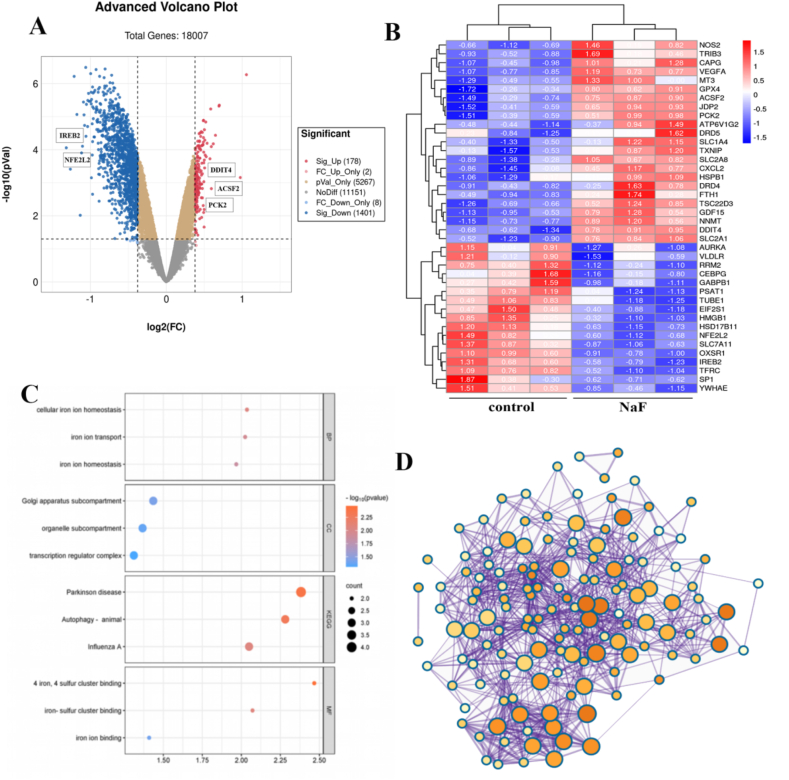
**Identifying dysregulated genes related to ferroptosis in fluoride-treated HOS cells** (A) Volcano plot displayed differentially expressed genes (DEGs) identified in GSE70719, with 178 upregulated genes represented by red points and 1401 downregulated genes by blue points. (B) Heatmap visualized the expression level of 40 dysregulated genes related to ferroptosis in controls and NaF-treated cells. (C) Bubble plots described the significantly enriched GO and KEGG terms. (D) PPI network identified 15 hub genes with complex modulation relations.

With an adjusted *P* value < 0.05 and |FC| > 1.3, we obtained 1579 DEGs from the GSE70719 dataset. Volcano plots were used to visualize the DEGs in Fig. 1A, in which 178 genes were upregulated and 1401 genes were downregulated. To investigate the potential involvement of ferroptosis in fluoride-induced cell injury, we then intersected these DEGs with 111 ferroptosis-related genes from the FerrDb database. 40 DEGs associated with ferroptosis were obtained, and their expression levels of which displayed in the heat map in Fig. 1B. The functions of these 40 ferroptosis-related DEGs were annotated using GO and KEGG enrichment analyses. This is illustrated by the bubble plots in Fig. 1C. Notably, we found significant enrichment GO terms related to iron ion homeostasis and binding, as well as KEGG pathways associated with neurodegenerative diseases such as Parkinson's disease. Additionally, we conducted a protein-protein interaction (PPI) network analysis using the REACTOME online database and depicted the network using Cytoscape software, as shown in Fig. 1D. We identified 15 highly interconnected hub DEGs, highlighting their potential importance in fluoride-induced cell injury and ferroptosis.

### 3.2 Fluoride can induce spinal cord injury with aberrant biomarkers

**Fig. 2:**
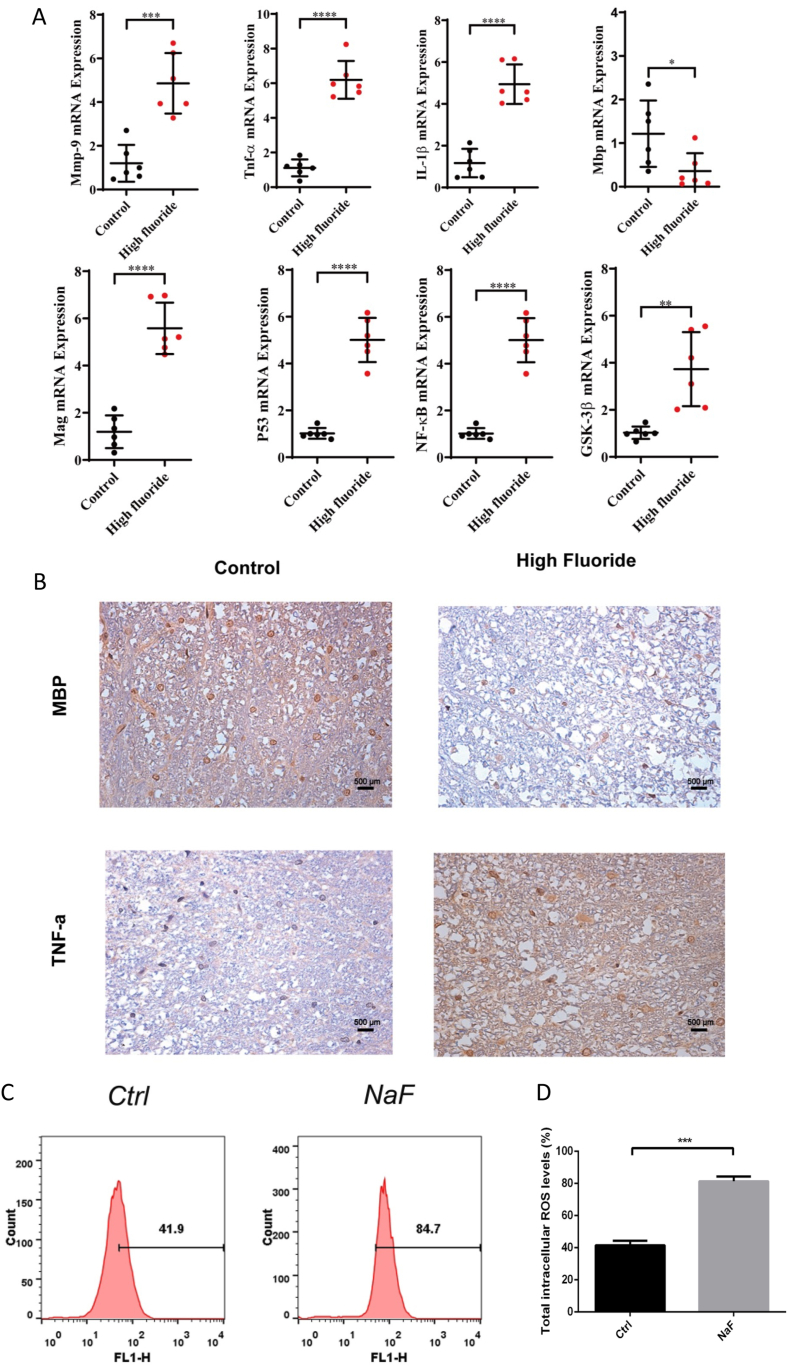
**Fluoride exposure induced spinal cord injury with aberrant biomarkers** (A) Scatter plots exhibited the relative mRNA expression levels of Mmp9, TNF-α, IL-1β, Mbp, Mag, P53, NF-κB, GSK-3β in controls and NaF-treated rats. (B) The IHC staining visualized the expression of TNF-α, MBP in controls and NaF-treated rats. (C–D) ROS content in the control and NaF-treated groups measured by flow cytometry. The numbers in the flow cytometry histograms represent the percentage of cells with elevated intracellular ROS levels above a specific threshold. Magnification: × 200, scale bars = 50 μm. Data are represented as the mean ± SEM (n = 6). ∗*P* < 0.05, ∗∗*P* < 0.01, ∗∗∗*P* < 0.001, ∗∗∗∗*P* < 0.0001.

To investigate the impact of fluoride on the spinal cord, we analyzed the mRNA levels of fluorosis-related inflammation indicators (Mmp9, GSK3β, TNF-α, IL-1β, P53, NF-κB, Mag, and Mbp) using qPCR. The results demonstrated a significant increase in the mRNA levels of Mmp9, TNF-α, IL-1β, Mag, P53, NF-κB, and GSK3β in the spinal cord tissues of rats exposed to NaF, in comparison to the control group. Conversely, mRNA levels of Mbp, a major component of the myelin sheath of Schwann cells and oligodendrocytes in the nervous system, decreased (Fig. 2A). Furthermore, our histopathological analysis showed a significant increase in TNF-α expression and a significant decrease in MBP in the fluoride-exposed group (Fig. 2B), indicating severe spinal cord injuries in the rats exposed to fluride. In addition, the ELISA experiment also demonstrated a significant increase in TNF-α expression in the group exposed to fluoride (Fig. S2A). The ROS assay results indicated a significant enhancement of ROS in the NAF group compared to the control group (Fig. 2C and D).

### 3.3 Ferroptosis is involved in spinal cord injury in rats exposed to fluoride

**Fig. 3:**
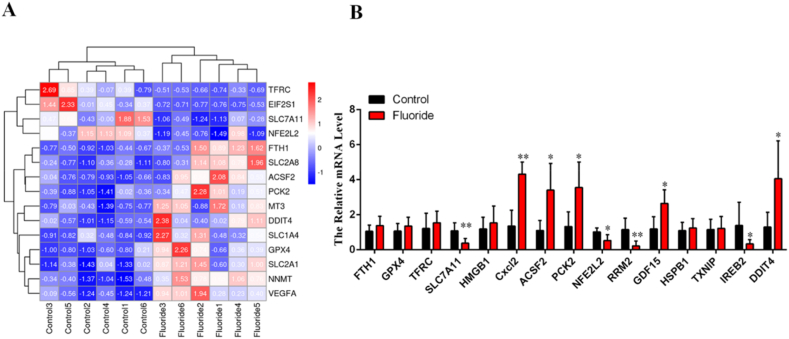
**Ferroptosis is involved in spinal cord injury in rats exposed to fluoride** (A) Heatmap represented the expression level of 15 hub genes associated with ferroptosis in 6 control rats and 6 NaF-treated rats. (B) Bar graph illustrated the expression of 15 hub genes associated with ferroptosis at group level.

In our previous study, we identified 15 hub genes that were dysregulated in fluoride-treated HOS cells and were associated with ferroptosis. Based on information from the FerrDb database, we categorized seven ferroptosis driver genes (TFRC, GDF15, HMGB1, CXCL2, ACSF2, PCK2, and DDIT4) and eight ferroptosis suppressor (FTH1, GPX4, SLC7A11, RRM2, NFE2L2, HSPB1, IREB2, and TXNIP). Subsequently, we measured the relative mRNA levels of these 15 hub genes in the spinal cord tissue of six control and six NaF-treated rats. The heatmap in Fig. 3A displays the relative expression levels of the 15 hub genes at the individual level. Notably, at the group level, the expression of ferroptosis driver genes, including CXCL2, DDIT4, GDF15, ACSF2, and PCK2 was significantly increased, whereas that of ferroptosis suppressor genes, including NFE2L2, SLC7A11, RRM2, and IREB2, was significantly downregulated (Fig. 3B).

### 3.4 Tanshinone ⅡA ameliorated spinal cord injury in rats exposed to fluoride

**Fig. 4:**
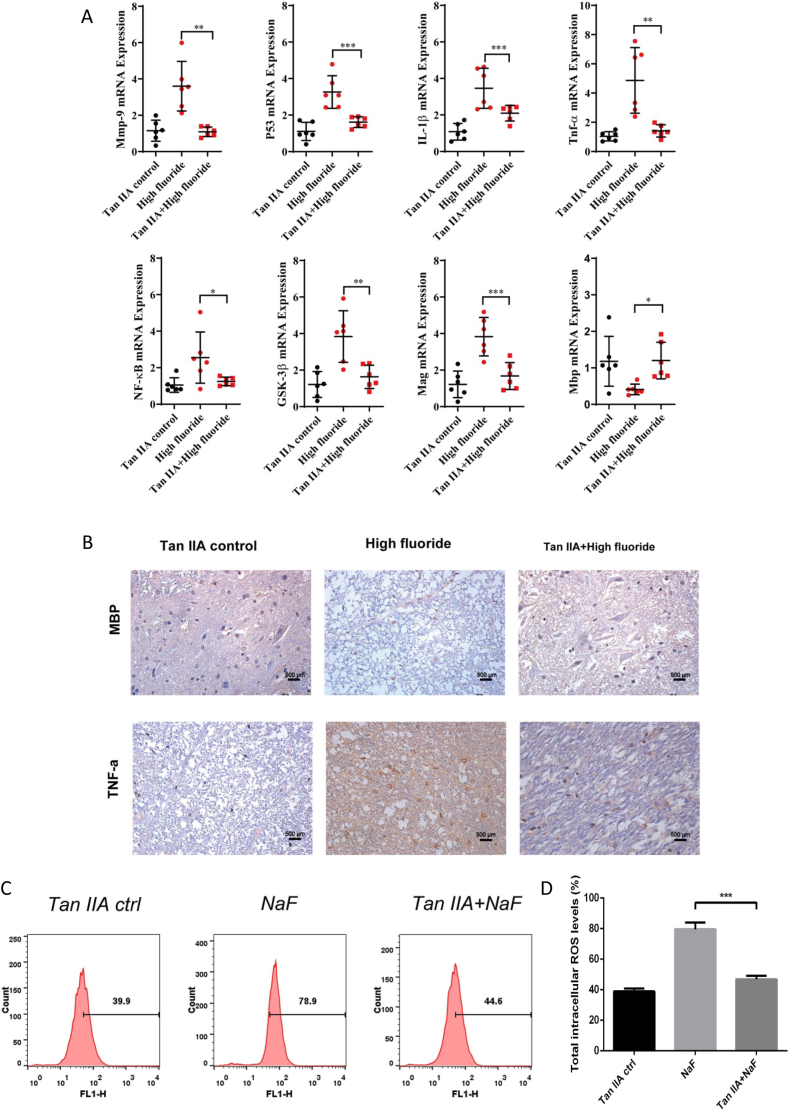
**The effect of TanⅡ on the fluoride-exposed rats** (A) Scatter plots displayed the relative mRNA expression levels of Mmp9, TNF-α, IL-1β, Mbp, Mag, P53, NF-κB, GSK-3β in Tan IIA control, high fluoride group, and Tan IIA + high fluoride group. (B) The IHC staining illustrated the expression of TNF-α and MBP in Tan IIA control, high fluoride group, and Tan IIA + high fluoride group. (C–D) ROS content in Tan IIA control, high fluoride group, and Tan IIA + high fluoride group measured by flow cytometry. The numbers in the flow cytometry histograms represent the percentage of cells with elevated intracellular ROS levels above a specific threshold. Magnification: × 200, scale bars = 50 μm. Data are represented as the mean ± SEM (n = 6). ∗*P* < 0.05, ∗∗*P* < 0.01, ∗∗∗*P* < 0.001, ∗∗∗∗*P* < 0.0001.

In the present study, a dose of 20 mg/(kg•d) Tan IIA can effectively restore aberrant inflammation and spinal cord injury biomarkers caused by fluoride. As is shown in Fig. 4A, the mRNA levels of Mmp9, TNF-α, Mag, IL-1β, P53, NF-κB, and GSK3β were significantly decreased, and the Mbp level was rescued in the Tanshinone IIA + high fluoride group compared to the high fluoride group. No significant changes were observed between the Tan IIA control and Tanshinone IIA + high-fluoride groups. Histopathological analysis of spinal cord tissues indicated that Tan IIA treatment reduced TNF-α expression and accelerated MBP expression (Fig. 4B). In addition, the ELISA experiment also demonstrated that Tan IIA treatment reduced TNF-α expression (Fig. S2B). The ROS assay results indicated a significant enhancement of ROS in the NAF group compared to the control group, and Tan IIA was able to reverse this phenomenon (Fig. 4C and D). Furthermore, no statistically significant alterations were detected in the mRNA levels of the 15 ferroptosis related genes(Fig. S1A), and 8 fluorosis-related inflammation indicators, including Mmp9, TNF-α, Mag, IL-1β, P53, NF-κB, and GSK3β when comparing the Tan IIA control group with the control group (Fig. S1B). Moreover, there were no notable histopathological changes in the expression of TNF-α and MBP observed in the spinal cord tissues (Fig. S1C).

### 3.5 Inhibition of ferroptosis and inflammatory by Tanshinone ⅡA improved spinal cord injury induced by fluoride

**Fig. 5:**
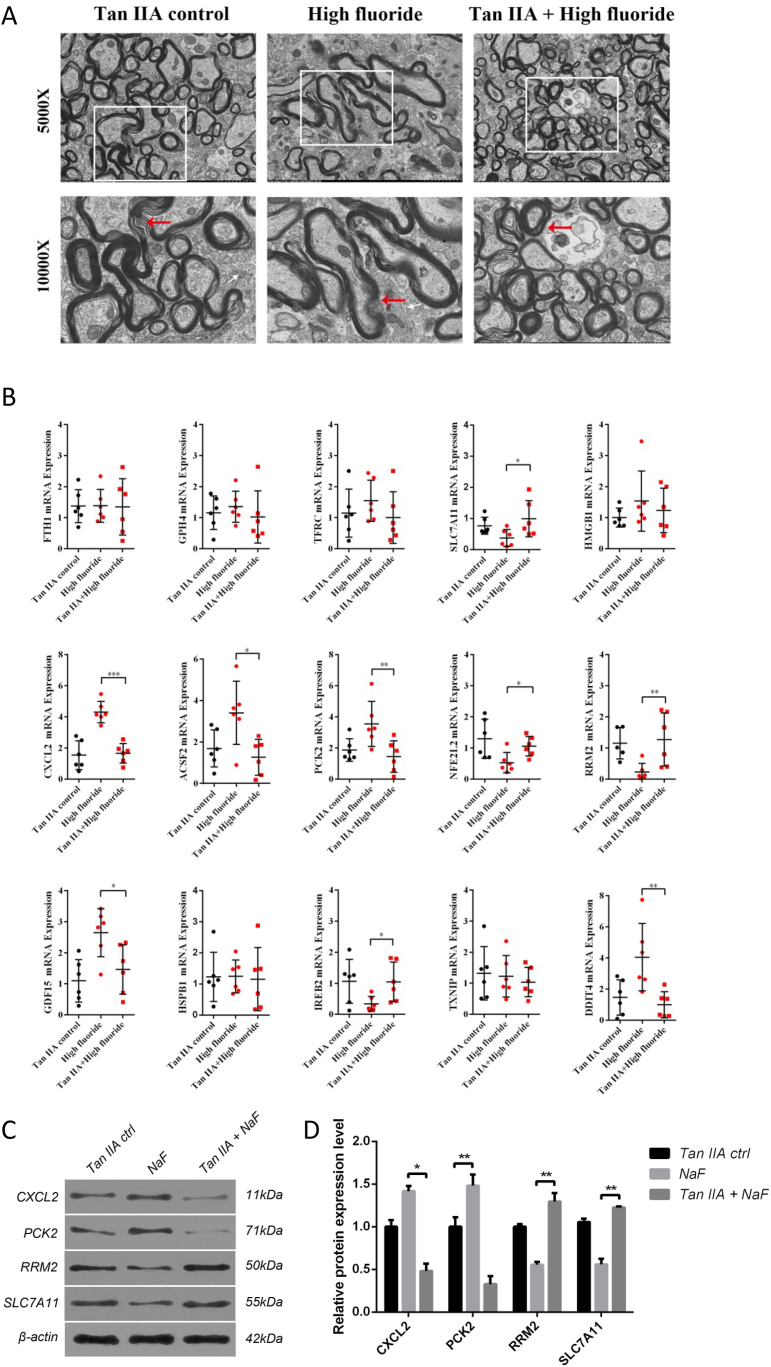
Tanshinone IIA improved spinal cord injury by inhibiting ferroptosis (A) Photomicrographs of spinal cord sections from Tanshinone IIA control, fluoride-exposed rats, and Tanshinone IIA + high fluoride group visualized by transmission electron microscopy (5000X, white box). Fibrous myelin integrity (indicated by red arrows) is maintained in both Tanshinone IIA control and Tanshinone IIA-treated groups, while in the fluorosis group, it appears loosely arranged with an increased gap and disordered structure. (B) Scatter plot exhibited the expression of 15 ferroptosis-related genes in Tan IIA control, high fluoride group, and Tan IIA + high fluoride group. (C–D) Western blots showed that Tan IIA treatment restored the reduced protein expression of SLC7A11 and RRM2 observed in the NaF group. Additionally, Tan IIA treatment decreased the elevated protein levels of CXCL2 and PCK2 in the NaF group. Uncropped and unadjusted original images of the Western blots are provided in Fig. S3. Data are represented as the mean ± SEM (n = 6). ∗P < 0.05, ∗∗P < 0.01, ∗∗∗P < 0.001, ∗∗∗∗P < 0.0001.

This study suggests that Tan IIA effective in reducing inflammation and spinal cord injury biomarkers in fluoride-treated rats. To assess structural changes, we examined fibrous myelin using transmission electron microscopy. Our findings showed that fibrous myelin remained intact in both the Tan IIA control and Tan IIA-treated groups, whereas in the fluorosis group, it was loosely arranged and exhibited an increased gap with a disordered structure (Fig. 5A). Furthermore, we investigated the effect of Tan IIA treatment on ferroptosis by measuring the expression levels of 15 ferroptosis-related hub genes. As illustrated in Fig. 5B, the mRNA levels of ferroptosis depressor genes (NFE2L2, SLC7A11, RRM2, and IREB2) were significantly increased in the Tan IIA group, whereas the expression levels of genes related to inflammation and apoptosis (CXCL2, DDIT4, GDF15, ACSF2, and PCK2) were significantly decreased. Additionally, we confirmed the expression of four significantly dysregulated genes at the protein level, including SLC7A11, RRM2, CXCL2, and PCK2 (Fig. 5C and D). The diminished expression of SLC7A11 and RRM2 in the NaF group was restored by Tan IIA treatment, while the heightened expression of CXCL2 and PCK2 was attenuated. These results indicate that Tan IIA may alleviate spinal cord injury caused by fluorosis by attenuating ferroptosis and inflammatory responses.

## 4 Discussion

Several studies have established a correlation between chronic exposure to inorganic fluorides and neurotoxicity. The mechanisms underlying fluoride-induced neurotoxicity include the disruption of synaptic plasticity and transmission, premature neuronal death, altered intracellular signaling, disrupted protein synthesis, loss of transcription and neurotrophic factors, oxidative stress, inflammatory processes, and metabolic changes [25,26]. However, the molecular mechanisms responsible for spinal cord injuries resulting from fluorosis remain elusive. In this study, we demonstrated that fluorosis can induce spinal cord injury from both molecular and structural perspectives, accompanied by the dysregulation of ferroptosis-related genes. Tan IIA can alleviate spinal cord injury caused by fluorosis by restoring dysregulated ferroptosis-related genes and SCI biomarkers of spinal cord injury.

Previous research demonstrated that chronic fluorosis induces spinal cord neuronal apoptosis via upregulation of P53 and MMP-9 expression in rats and that short-term defluorination did not alleviate the overexpression of these markers [9]. Moreover, chronic nerve tissue injury can lead to increased P53 levels, resulting in apoptosis, while high MMP-9 expression can increase capillary permeability [27]. Fluorosis can directly damage the myelin nerve in the central brain of rats by causing MBP loss, which in turn leads to the breakdown of myelin structures due to the dissolution of myelin lipids [28]. In addition, various inflammatory indicators (TNF-α, IL-1β, NF-κB), and other fluorosis-related indicators (Mag, GSK3β) were also included to assess spinal cord injury in fluorosis rats [10,11]. In our study, we observed that excessive fluoride exposure can lead to the expression of inflammation and spinal cord injury indicators, such as Mmp9, TNF-α, IL-1β, Mag, and P53. Histopathological experiments showed that the mRNA level of Mbp, a major component of the myelin sheath of Schwann cells and oligodendrocytes in the nervous system, was decreased while the expression of TNF-α increased (Fig. 2A and B), indicating that fluorosis causes notable injury in the spinal cord.

Ferroptosis is a distinct form of regulated cell death that is separate from necrosis, apoptosis, and autophagy [29,30]. It is characterized by the depletion of unsaturated fatty acids in the plasma membrane and the accumulation of iron-induced lipid reactive oxygen species (ROS). ROS plays a key role in the process of ferroptosis by promoting lipid peroxidation, which exacerbates damage to the cell membrane and triggers ferroptosis. Additionally, iron generates ROS through the Fenton reaction, which accelerates the lipid peroxidation process, making this a core characteristic of ferroptosis [31]. Ferroptosis shares some features with other forms of cell death, such as caspase activation, mitochondrial cytochrome *c* release, and chromatin fragmentation [32]. Previous studies have established a link between ferroptosis and nerve injuries such as traumatic brain injury [33,34], ischemia-reperfusion injury [35], Parkinson's disease [36], intracerebral hemorrhage, and periventricular leukomalacia [36]. In the present study, differentially expressed genes associated with ferroptosis in Na-F cells were identified by intersection analysis of the GSE70719 dataset and ferroptosis-related genes from the FerrDb database, and 15 hub genes were selected based on the PPI network. The hub genes (TFRC, GDF15, HMGB1, FTH1, GPX4, SLC7A11, NFE2L2, HSPB1, CXCL2, ACSF2, PCK2, RRM2, IREB2, TXNIP, and DDIT4) were verified in fluoride-exposed rats. The expression levels of some important ferroptosis suppressor genes (SLC7A11, NFE2L2, IREB2, and RRM2) significantly decreased, whereas those of several ferroptosis driver genes, including CXCL2, DDIT4, GDF15, ACSF2, and PCK2, significantly increased. (Fig. 3). Nrf2 (transcription factor NFE2L2/NRF2) silencing can dramatically reduce SLC7A11 levels and exacerbate cellular injuries [37]. Quercetin (QCT) significantly inhibits SLC7A11 and GPX4 expression and ameliorates macrophage chemotaxis in acute kidney injury [38]. CXCL2, a proinflammatory cytokine, improves spinal cord injury via the activation of major inflammatory intracellular signaling activation [39]. GDF15 is overexpressed during intestinal ischemia/reperfusion Injury [40]. One study identified that DDIT4 impacts spinal cord injury based on a PPI network [41]. These findings suggest that fluoride exposure causes cell injury by promoting ferroptosis.

Tan IIA, also known as Tanshinone IIA, is a natural compound found in the roots of Salvia miltiorrhiza, a traditional Chinese herb that has been used for centuries to treat cardiovascular and cerebrovascular diseases. Many studies have reported that Tan IIA has multiple beneficial effects, including anti-inflammatory, anti-proliferative, anti-apoptotic, and antioxidant properties. In the context of cardiovascular health, Tan IIA protects against apoptosis and autophagy, inhibits thrombosis, reduces oxidative stress, and inhibits the expression of adhesion molecules involved in recruiting inflammatory cells to the vascular endothelium [[42], [43], [44], [45]] Additionally, Tan IIA supports the survival of neurons by reducing the amount of glial fibrillary acidic proteins in brain tissues [46]. It can also reduce neuroinflammation in models of neurodegenerative diseases such as Alzheimer's disease. More recently, it was reported that treatment with Tan IIA reduced the expression of proinflammatory cytokines such as NF-κB, TNF-α, IL-6, and IL-1β in Alzheimer's disease model [27,28]. In this study, we showed that Tan IIA protected against spinal cord injury induced by fluorosis at a dose of 20 mg/(kg•d). The mRNA levels of Mmp9, TNF-α, IL-1β, Mag, P53, NF-κB, and GSK3β were significantly reduced in the Tan IIA treatment groups compared to those in the NaF-stimulated rats. Moreover, the expression of ferroptosis depressor genes (SLC7A11, NFE2L2, IREB2, and RRM2) significantly increased, and the expression of ferroptosis driver genes (CXCL2, DDIT4, GDF15, ACSF2, and PCK2) significantly decreased in the Tan IIA-treated group (Fig. 4).

Our study investigated the effect of Tan IIA on fluoride-induced spinal cord injury as well as the underlying molecular mechanism. The present findings suggest that Tan IIA treatment significantly attenuates spinal cord injury by inhibiting inflammation and ferroptosis, which may provide a potential therapeutic strategy for spinal cord injury resulting from fluorosis. However, our study has several limitations. First,

This study was conducted using animal models, and further research is needed to investigate the therapeutic effects of Tan IIA on neurofluorosis in humans. Second, this study did not evaluate the long-term effects of Tan IIA treatment on spinal cord injury. Third, this study did not explore the optimal dosage and duration of Tan IIA treatment for spinal cord injury. These limitations should be considered when interpreting the results of this study and when designing future research.

### 4.1 Ethical Compliance

Research experiments conducted in this article with animals or humans were approved by the Ethical Committee and responsible authorities of our research organization(s) following all guidelines, regulations, legal, and ethical standards as required for humans or animals (approve number: 2020C08).

## CRediT authorship contribution statement

**Qingfeng Shen:** Resources, Project administration, Methodology, Investigation, Data curation, Conceptualization. **Shibo Ma:** Methodology, Investigation, Formal analysis, Data curation. **Lingbo Li:** Resources, Methodology, Investigation, Formal analysis, Data curation, Conceptualization. **Yingpeng Xia:** Writing – review & editing, Writing – original draft, Validation, Supervision, Resources, Project administration, Methodology, Investigation, Formal analysis, Data curation.

## Data availability statement

Raw data were generated at [Tianjin Union Medical Center]. Derived data supporting the findings of this study are available from the corresponding author [Y.X.] on request.

## Declaration of competing interest

The authors declare that they have no known competing financial interests or personal relationships that could have appeared to influence the work reported in this paper.
