# Supplementary figures and images for: Tanshinone IIA attenuates fluoride-induced spinal cord injury by inhibiting ferroptosis and inflammation
Source: Heliyon. 2024 Nov 28;10(23):e40549. doi: 10.1016/j.heliyon.2024.e40549 (PMC11648119; doi:10.1016/j.heliyon.2024.e40549)

**A**

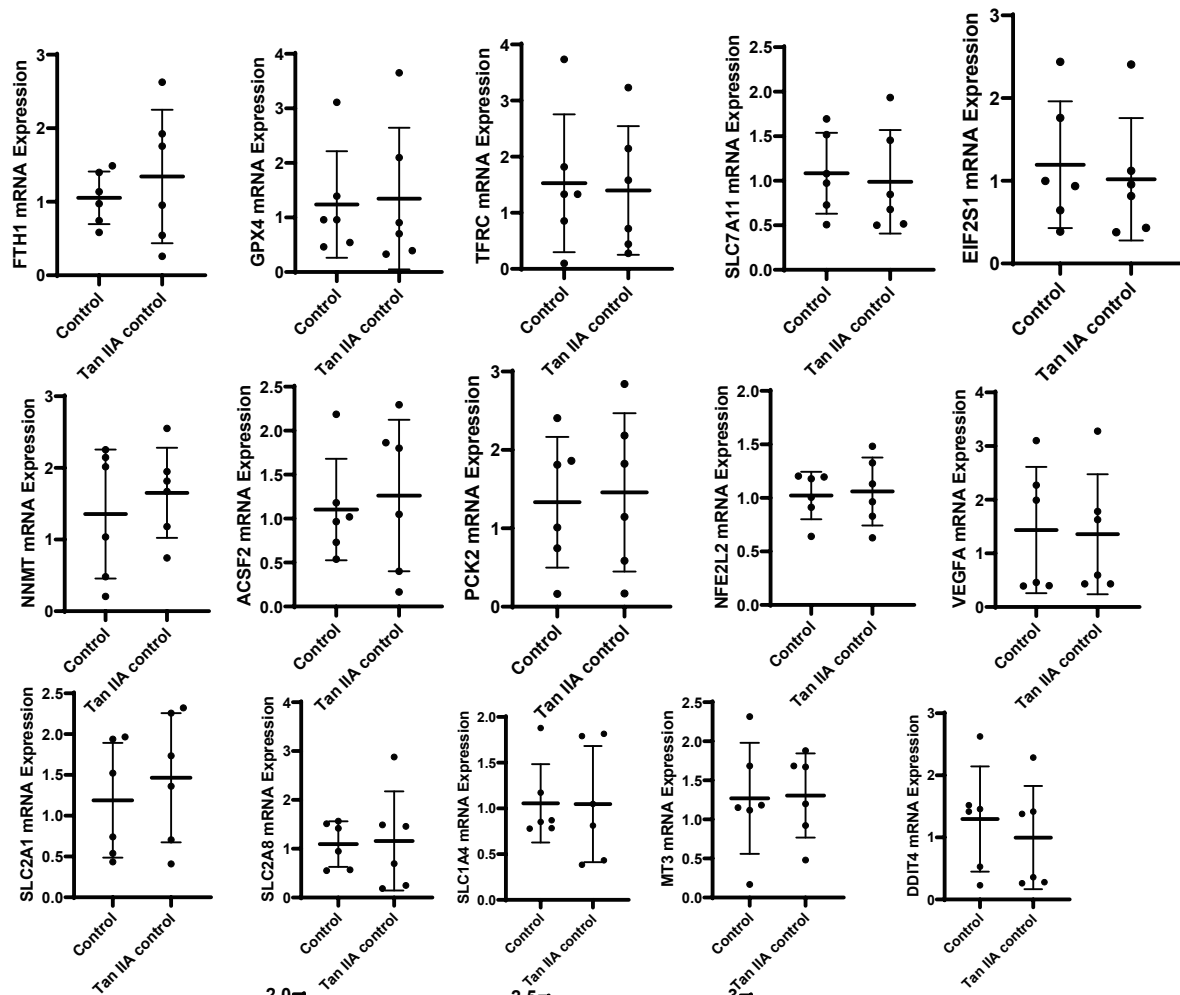

**B**

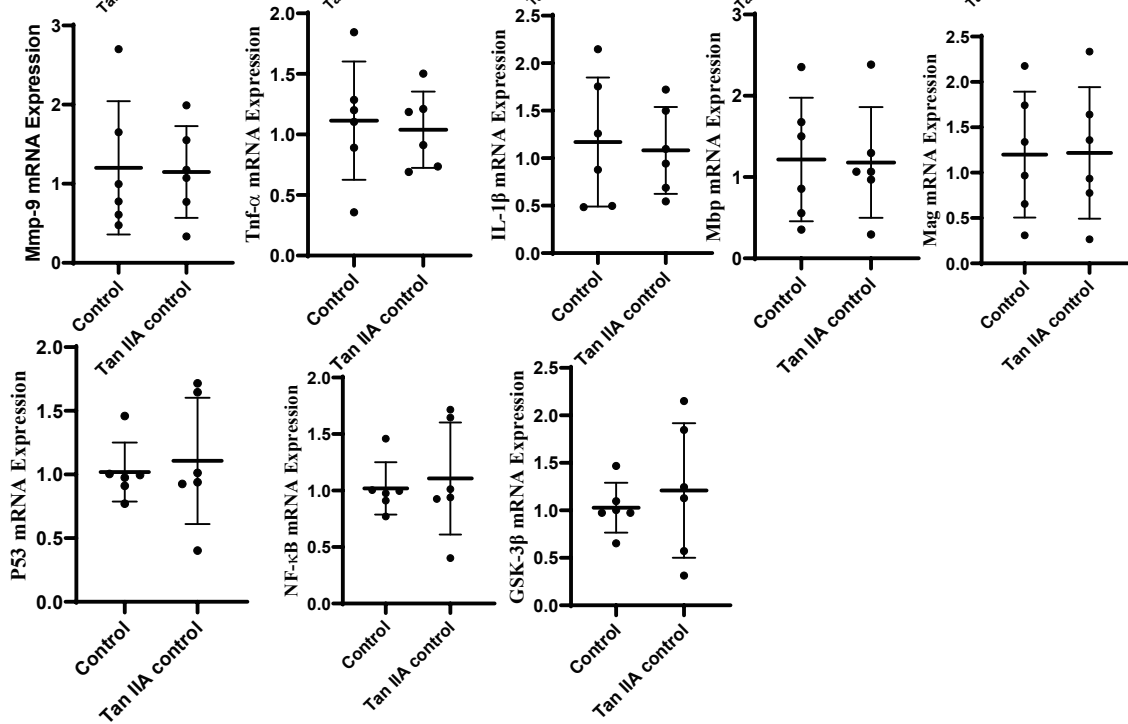

**C**

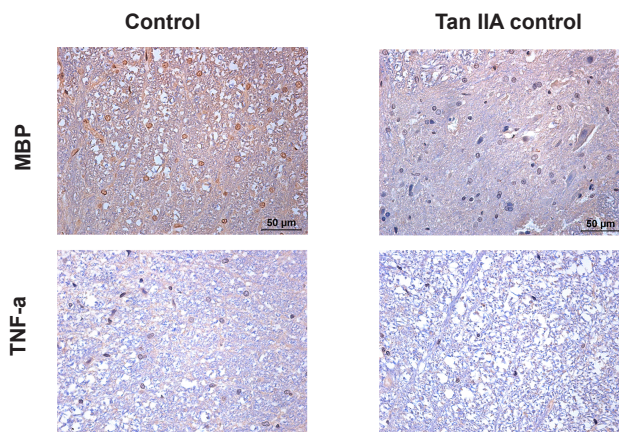

Supplement: Multimedia component 1 [file mmc1.pdf]

**CXCL2**

**
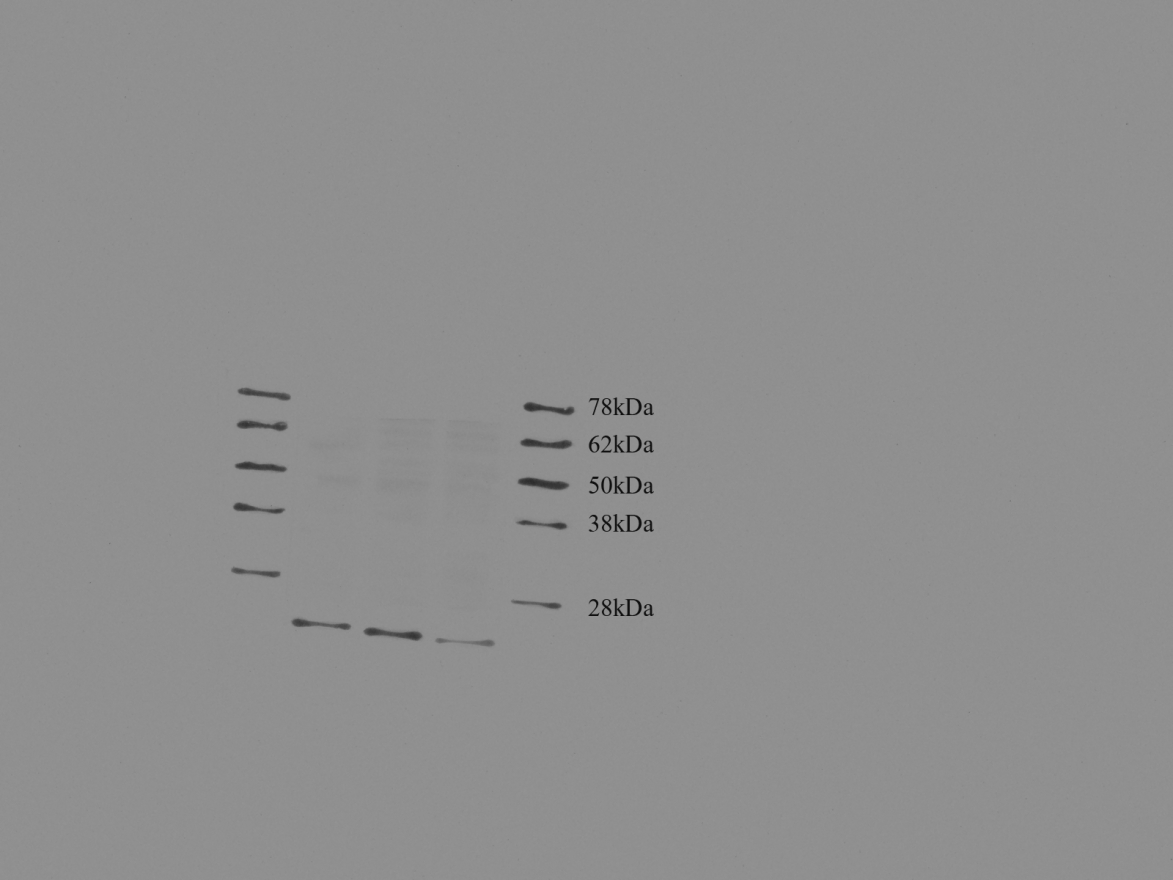
**

**PCK2**

**
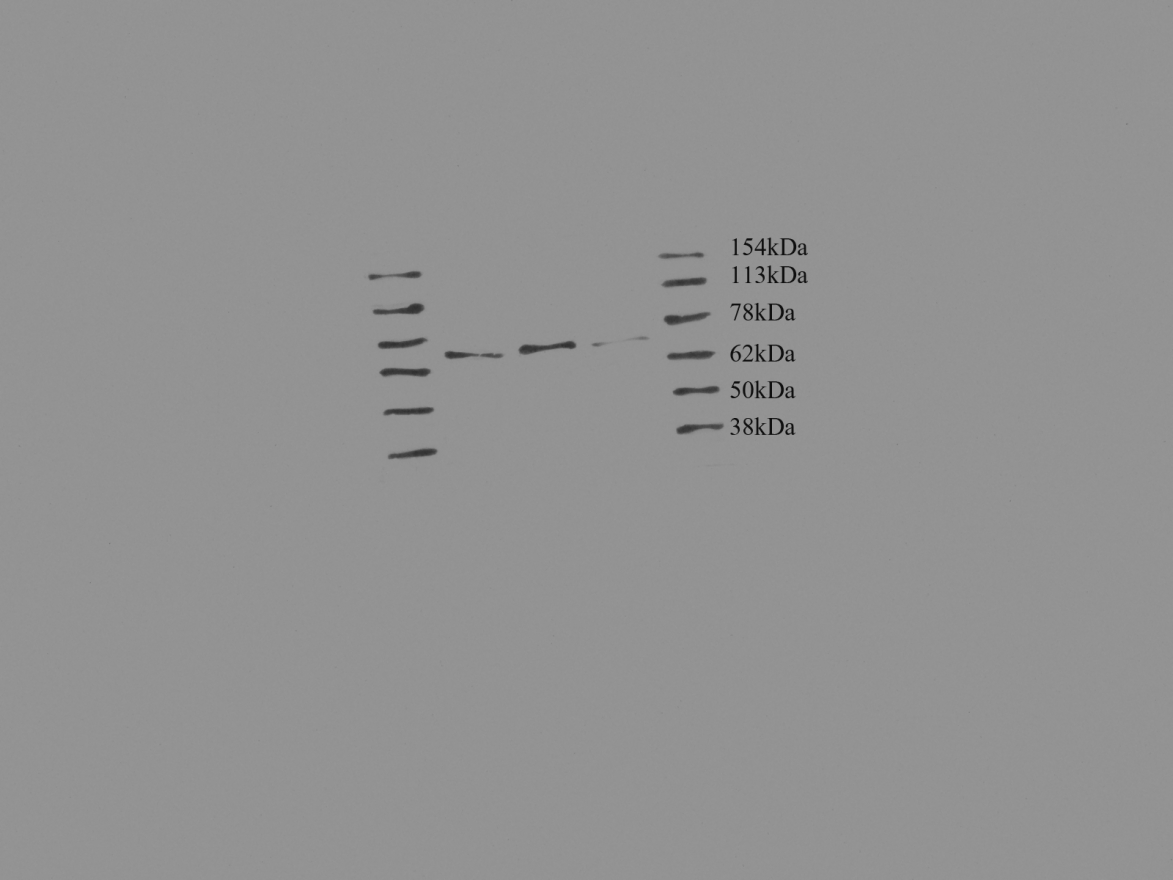
**

**RRM2**

**
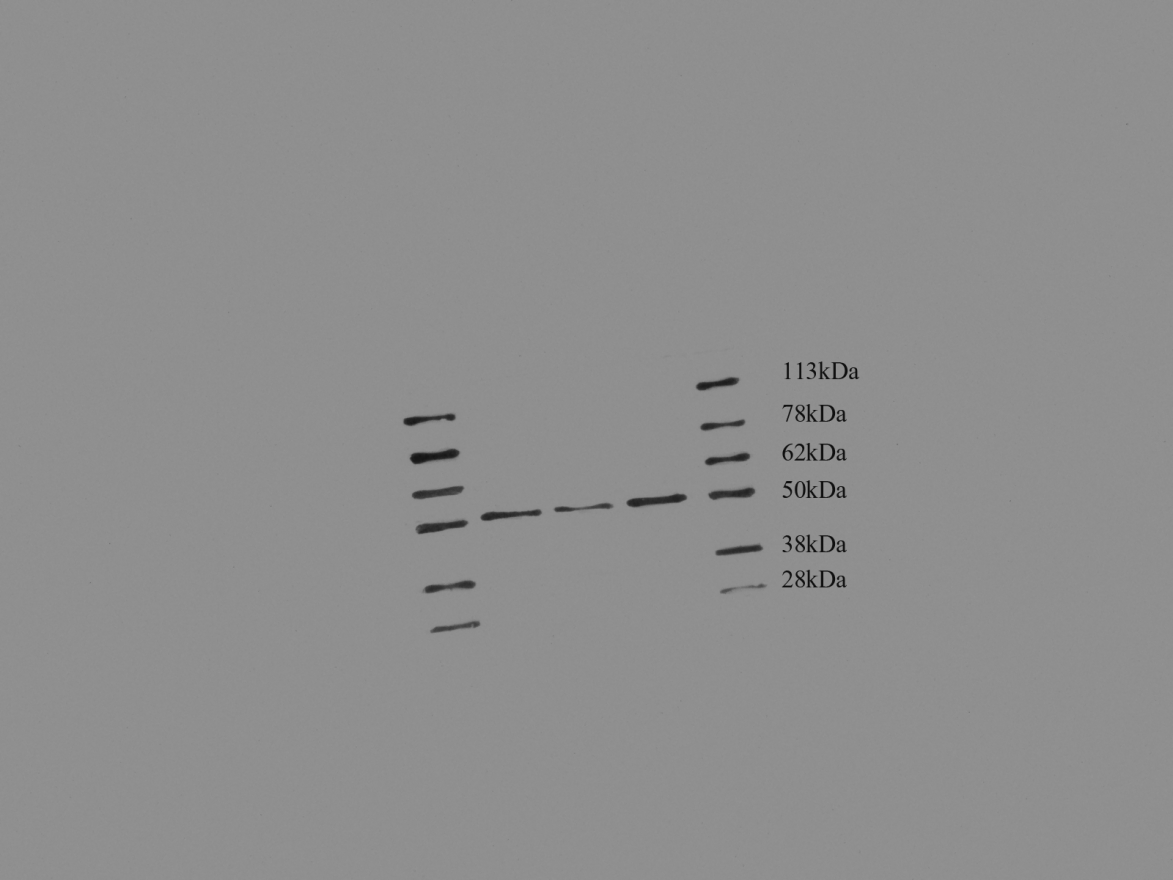
**

**SLC7A11**

**
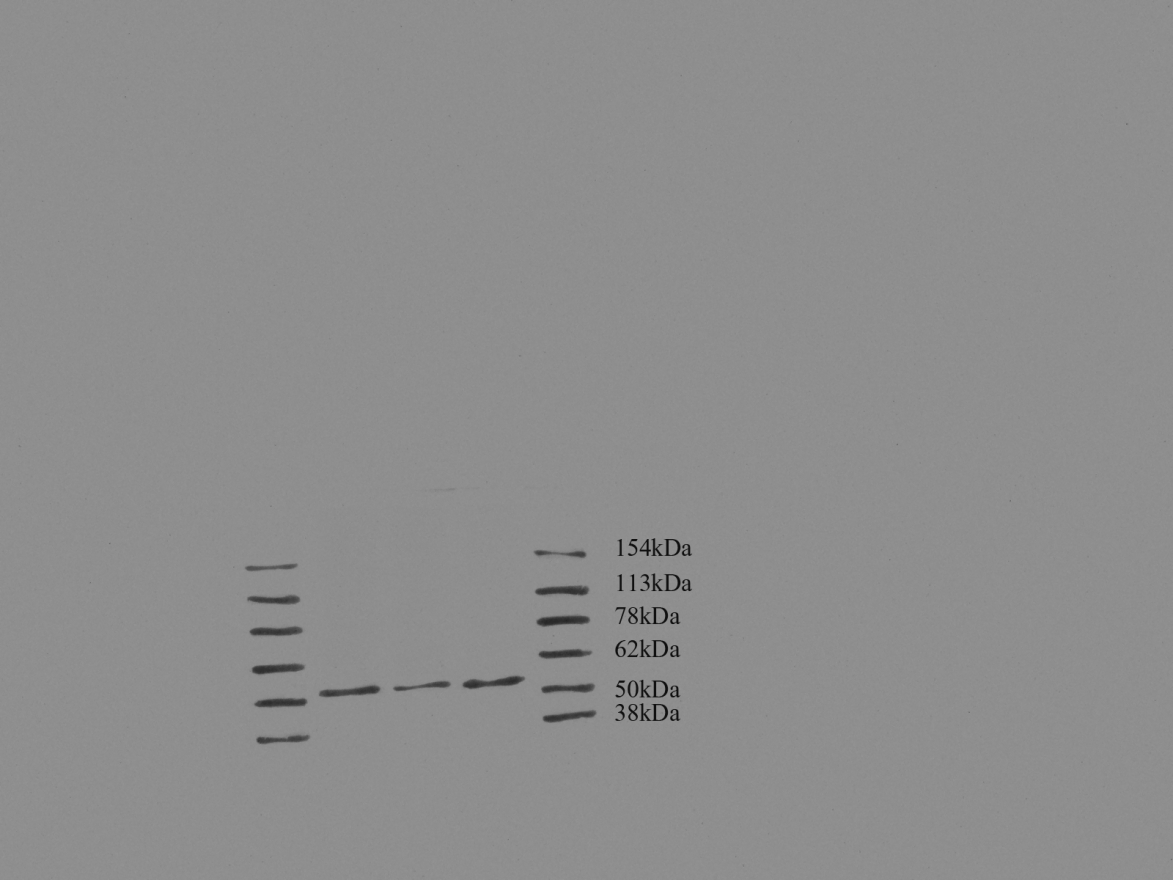
**

**β-actin**

**
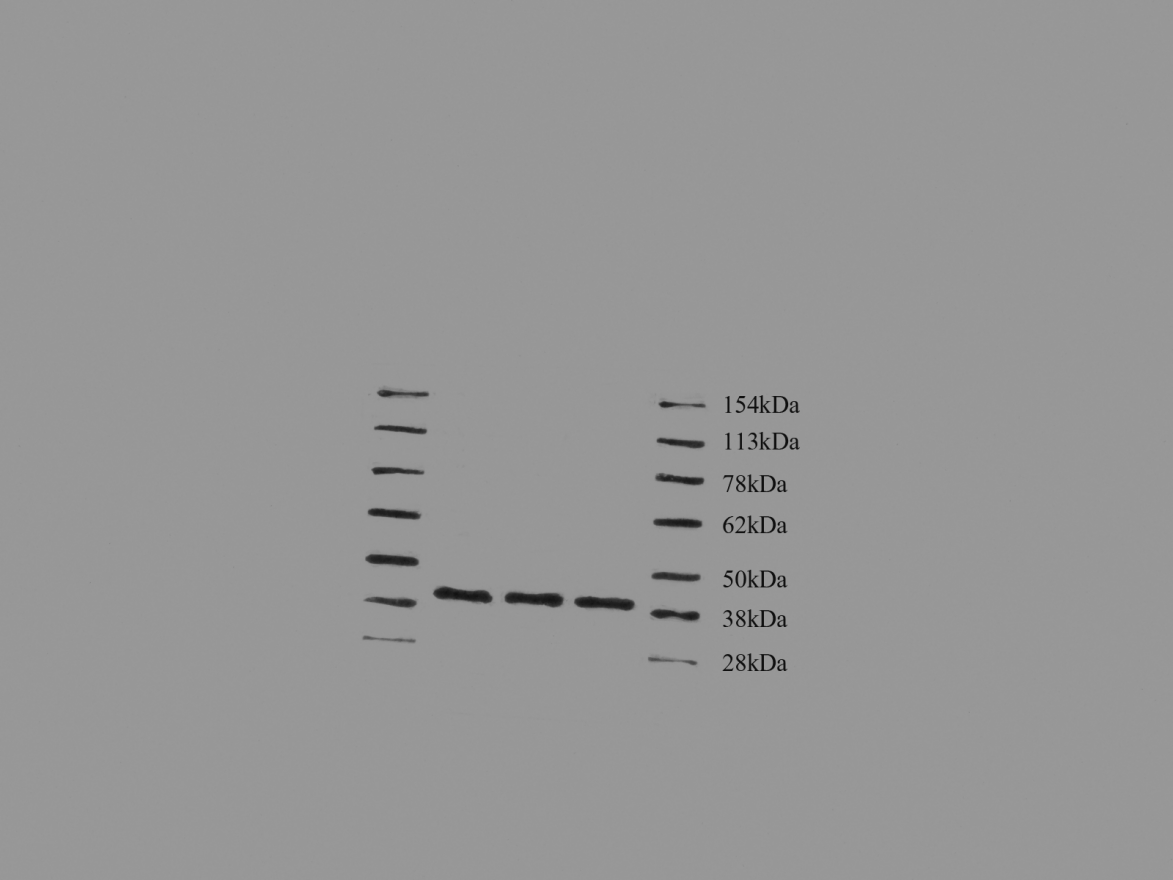
**

Supplement: Multimedia component 3 [file mmc3.docx]
