# Supplementary material for: Tanshinone IIA attenuates fluoride-induced spinal cord injury by inhibiting ferroptosis and inflammation
Source: Heliyon. 2024 Nov 28;10(23):e40549. doi: 10.1016/j.heliyon.2024.e40549 (PMC11648119; doi:10.1016/j.heliyon.2024.e40549)
Supplement: Multimedia component 2 [file mmc2.docx]

Figure S2. TNF-α ELISA experiment. (A) The results of the TNF-α ELISA experiment in controls and NaF-treated groups. (B)The results of the TNF-α ELISA experiment in Tan IIA control, high fluoride group, and Tan IIA + high fluoride group.
